# Management of Amiodarone-Induced Thyrotoxicosis at a Cardiac Transplantation Centre

**DOI:** 10.3389/fendo.2018.00482

**Published:** 2018-08-22

**Authors:** Michelle Isaacs, Monique Costin, Ron Bova, Helen L. Barrett, Drew Heffernan, Katherine Samaras, Jerry R. Greenfield

**Affiliations:** ^1^Department of Endocrinology, St Vincent's Hospital Sydney, Darlinghurst, NSW, Australia; ^2^Northern Sydney Endocrine Centre, Sydney, NSW, Australia; ^3^Faculty of Medicine, University of Notre Dame, Sydney, NSW, Australia; ^4^Faculty of Medicine, University of New South Wales, Sydney, NSW, Australia; ^5^Department of Ear Nose and Throat Surgery, St Vincent's Hospital Sydney, Darlinghurst, NSW, Australia; ^6^Department of Obstetric Medicine, Royal Brisbane and Women's Hospital, Brisbane, QLD, Australia; ^7^Faculty of Medicine, University of Queensland, Brisbane, QLD, Australia; ^8^Department of Anaesthetics, St Vincent's Hospital Sydney, Darlinghurst, NSW, Australia; ^9^Diabetes and Metabolism Division, Garvan Institute of Medical Research, Sydney, NSW, Australia

**Keywords:** amiodarone induced thyrotoxicosis, amiodarone, thyrotoxicosis, hyperthyroidism, thyroidectomy, heart failure

## Abstract

**Background:** Amiodarone-induced thyrotoxicosis (AIT) is associated with significant morbidity and mortality, particularly in patients with cardiac failure. The aim of the study was to evaluate the management of AIT at a tertiary hospital specialising in cardiac failure and transplantation.

**Methods:** Retrospective audit of 66 patients treated for AIT by Endocrinology (2007–2016), classified as type 1 (T1) or type 2 (T2) based on radiological criteria. Main outcome measurements were response rate to initial treatment, time to euthyroidism, and frequency/safety of thyroidectomy.

**Results:** Mean age was 60 ± 2 years; 80% were male. Sixty-four patients commenced medical treatment: thionamides (THIO) in 23, glucocorticoids (GC) in 17 and combination (COMB) in 24. Median thyroxine (fT4) was 35.1 (31.2–46.7) in THIO, 43.1 (30.4 –60.7) in GC, and 60.0 (39.0 –>99.9) pmol/L in COMB (*p* = 0.01). Initial therapy induced euthyroidism in 52%: 70% THIO, 53% GC, and 33% COMB (*p* = 0.045) by 100 (49–167), 47 (35–61), and 53 (45–99) days, respectively (*p* = 0.02). A further 11% became euthyroid after transitioning from monotherapy to COMB. Thyroidectomy was undertaken in 33%. Patients who underwent thyroidectomy were younger (54 ± 3 vs. 63 ± 2 years; *p* = 0.03), with higher prevalence of severely impaired left ventricular function prior to diagnosis of AIT (38 vs. 18%; *p* = 0.08). Despite median American Society of Anaesthesiologists classification 4, no thyroidectomy patient experienced cardiorespiratory complications/death.

**Conclusions:** Patients with AIT had limited response to medical treatment. The poorest response was observed in COMB group, likely related to greater hyperthyroidism severity. Thyroidectomy is safe in patients with severe cardiac failure if performed in a centre with cardiac anaesthetic expertise. There should be low threshold for proceeding to thyroidectomy in patients with severe AIT and/or cardiac failure.

## Introduction

Amiodarone is a class 3 anti-arrhythmic agent that increases the myocardial refractory period by inhibiting Na-K ATPase activity (1). It restores sinus rhythm in one-third of patients with chronic atrial fibrillation and reduces recurrence by 81% (2, 3). It is also effective for ventricular tachyarrhythmias and, in select patients, for prevention of sudden cardiac death (1).

Amiodarone's adverse effects include pulmonary toxicity, hepatitis and thyroid dysfunction (thyrotoxicosis or hypothyroidism) (4, 5). Type 1 amiodarone-induced thyrotoxicosis (T1 AIT) results from excessive thyroid hormone synthesis due to iodine loading (iodine constitutes one-third of amiodarone's weight); this typically occurs in an underlying nodular goitre or in latent Graves' disease (1, 5, 6). In contrast, type 2 (T2) is a destructive thyroiditis that may occur in an otherwise normal gland (5, 6). T1 is traditionally treated with thionamides and T2 with glucocorticoids (5, 6). However, this classification is likely to be an oversimplification, given that patients often fail to respond to therapy directed against their specific subtype (7). T2 can occur within a goitre, such that mixed/indefinite forms of AIT exist in clinical practice (6, 8). Mixed/indefinite forms are not well characterised and diagnosis is often made retrospectively following a trial of therapy (6).

AIT is associated with significant cardiac morbidity and mortality (9), particularly in patients with impaired left ventricular function (10, 11). Prompt and effective restoration of euthyroidism is of paramount importance in cardiac failure patients but may be difficult to achieve with medication. For instance, treatment of T1 often typically requires a prolonged course of thionamides (6). Further, AIT may be refractory even to combination glucocorticoid and thionamide therapy (12–15). Emergency thyroidectomy rapidly restores euthyroidism and improves left ventricular ejection fraction (LVEF) in those with moderate-severe systolic dysfunction (16). Importantly, it allows amiodarone retreatment. The American Thyroid Association (ATA) recommends thyroidectomy for cases of AIT unresponsive to medical therapy (7). A recently published guideline from the European Thyroid Association additionally recommends thyroidectomy in patients with severe underlying cardiac disease or deteriorating cardiac function (6).

The aim of this study was to evaluate the management of AIT at St Vincent's Hospital, the NSW cardiac transplantation centre, including the frequency and safety of total thyroidectomy in patients with cardiac failure.

## Materials and methods

### Study design

This was a retrospective study of AIT patients at St Vincent's Hospital, Sydney between January 2007 and December 2016. Patients were identified by searching medical records (ICD-10 codes E05.8 and E06.4) or direct notification by the treating Endocrinologist. AIT diagnostic criteria were: suppressed thyroid-stimulating hormone (TSH), free thyroxine (fT4) above the upper limit of the assay reference interval, and amiodarone use within the preceding 12 months. Of the 73 patients 7 were excluded due to no follow-up; data for 66 were included in analyses.

For most patients, thyroid function tests were measured by Roche electrochemiluminescent assay (Roche Diagnostics, NSW, Australia); reference intervals were: TSH 0.4–4.2 mIU/L, fT4 11.0–22.0 pmol/L, fT3 3.0–6.2 pmol/L. Where TSH and fT4 were measured by Architect i2000 (Abbott Diagnostics, NSW, Australia) or Advia Centaur (Siemens Healthcare, NSW, Australia) immunoassays, the results were transformed to give values comparable to those that would be obtained by the Roche assay (17). Only fT3 results measured by the Roche assay were reported as data regarding comparable values on the other two platforms was absent.

Patients' cardiac comorbidities were determined from documentation by their Cardiologist. In patients with a history of cardiac failure, the severity of left ventricular dysfunction was determined by transthoracic echocardiographic measurement of LVEF in the 12 months prior to AIT diagnosis (normal LVEF = 50–70%, mild = 40–49%, moderate = 30–39%, severe <30%) (18). Duration of amiodarone treatment was established from the medical record; in some cases the documented duration was based on patient recall. Patients were classified as having T1 or T2 AIT by a single investigator (MI) based on reported imaging criteria (6). T1 criteria were gland hypervascularity on colour flow Doppler sonography (CFDS), grey-scale sonographic evidence of thyromegaly (moderate or greater) and/or at least one nodule ≥10 mm, or normal or increased scintigraphic uptake. T2 criteria were absence of all of these features. Resolution of AIT was ascertained by the time to euthyroidism, defined as TSH within the reference interval in patients on medical treatment, or time of thyroidectomy.

Thyroidectomy was performed by the same surgeon (RB) and the same anaesthetist (DH) in most cases. All patients had general anaesthesia consisting of propofol and fentanyl induction. Muscle relaxant was given for insertion of a Nerve Integrity Monitoring endotracheal tube under videolaryngoscopic control. An arterial line was placed in all patients in addition to routine anaesthetic monitoring of ECG, pulse oximetry, anaesthetic gases and carbon dioxide. Anaesthetic maintenance was with a volatile anaesthetic agent, usually sevoflurane and a remifentanil infusion. No further muscle relaxants were given to enable monitoring of recurrent laryngeal nerve function and all patients were extubated at the end of the surgery. All patients were transferred to the Intensive Care Unit for post-operative monitoring.

### Ethics

This study was approved by the St Vincent's Hospital Human Research Ethics Committee.

### Statistics

Numerical data were expressed as mean ± standard error, or median (interquartile range) for normal and non-normal data, respectively; categorical data were expressed as count (%). Non-normal numerical data were log-transformed, except where data was censored (laboratory values above or below the detection limit of the assay), in which case non-parametric analyses were performed. Differences between treatment groups were tested with ANOVA (*post-hoc* Tukey test) or Kruskall–Wallis (*post-hoc* Dunn's test) for numerical data, or Fischer Exact or Chi-squared tests for categorical data. Differences between patients who achieved euthyroidism with medication and those who required thyroidectomy were analysed with the Student's *t*-test or Mann–Whitney test for numerical data, and Fischer Exact test for categorical data. *P* < 0.05 was considered statistically significant. Analyses were performed with GraphPad Prism (Version 7.0, GraphPad Software, La Jolla California USA).

## Results

### Patient characteristics and initial management of AIT

Patient characteristics are shown in Table 1. Mean age was 60 ± 2 years; 80% were male. Duration of amiodarone therapy prior to development of AIT was 11 (2–36) months in T1 and 24 (12–31) months in T2 (*p* = 0.19). Twenty-four (36%) of patients presented with cardiac symptoms: 14 with decompensated heart failure, 13 with arrhythmias, and 1 with angina.

**Table 1:** Characteristics of patients at time of diagnosis of AIT and initial medication doses.

	**COMB (*n =* 24)**	**GC (*n =* 17)**	**THIO (*n =* 23)**	***p*-value**
Age (years)	55.9 ± 3.2	59.8 ± 3.1	63.4 ± 3.6	0.27
Male	19 (79%)	13 (76%)	20 (87%)	0.82
Arrhythmia history Atrial Ventricular Both	13 (54%) 2 (8%) 9 (38%)	12 (71%) 0 (0%) 5 (29%)	17 (74%) 3 (13%) 3 (13%)	NC
Ischaemic heart disease	7 (29%)	4 (24%)	8 (35%)	0.74
Cardiac failure	17 (71%)	10 (59%)	14 (61%)	0.68
LVEF <30%	7 (29%)	4 (24%)	4 (17%)	0.66
Cardiac transplant	3 (13%)	3 (18%)	1 (4%)	0.41
TSH <0.05 mIU/L	22 (92%)	14 (82%)	19 (82%)	0.59
fT4 (pmol/L)	60.0 (39.0 –>99.9)^*†*^	43.1 (30.4–60.7)	35.1 (31.2–46.7)^*‡*^	0.01
fT3 (pmol/L) (*n* = 40)	7.9 (6.3 –14.0)	6.2 (4.7–8.7)	6.4 (4.0–9.4)	0.24
Type of AIT 1 2	6 (25%)^§^ 13 (54%)	5 (29%) 12 (71%)	10 (43%) 13 (57%)	0.59
Prednisone (mg/d)	40 (30–50)	40 (35–50)	NA	NA
Carbimazole (mg/d)	32.8 ± 2.7^*†*^ (*n* = 16)	NA	21.2 ± 2.5^*‡*^ (*n* = 21)	NA
Propylthiouracil (mg/d)	400 (225.0–437.5) (*n* = 8)	NA	550 (300.0–800.0) (*n* = 2)	NA

† p < 0.05 compared to THIO;

‡ *p < 0.05 compared to COMB*;

§ *total ≠ 100% as 5 patients treated with COMB could not be classified as T1 or T2, NC, not calculated; NA, not applicable*.

Conventional thyroid ultrasound detected thyromegaly and/or nodular disease in 22 of 61 patients. Hypervascular CFDS pattern was present in 7 of 52 patients. Thirty-seven patients had assessment of thyroid uptake by ^99*m*^Technetium pertechnetate scan; all but 4 had decreased or no uptake. TSH receptor antibodies were negative in the 47 patients tested; thyroid peroxidase/microsomal antibodies were positive in 3/58 (5%). Overall, T1 prevalence was 31% (*n* = 21) and T2 59% (*n* = 40); the remaining 5 patients could not be classified due to lack of imaging.

Amiodarone was ceased in 63 patients (95%). In 2 asymptomatic patients with mild thyrotoxicosis, no additional therapy was required after amiodarone cessation and serial thyroid function tests showed resolution. Seventeen patients (26%) initially commenced glucocorticoids (GC), 23 (35%) thionamides (THIO), and 24 (36%) GC and THIO simultaneously or within 2 weeks of each other (COMB). No patient received perchlorate. Despite recommendations that patients with T1 AIT be treated with thionamides and T2 with glucocorticoids, the proportion of patients with T1 and T2 AIT did not differ between initial treatment groups. In many cases, initial treatment was commenced prior to investigation results being available. In 2 patients with T1, THIO were not prescribed as initial treatment due to neutropenia in the context of transplant.

Patients in COMB group had significantly higher fT4 at time of diagnosis than THIO (60.0 [39.0–>99.9] vs. 35.1 [31.2–46.7] pmol/L; *p* = 0.01) and were treated with higher initial doses of carbimazole (33 ± 3 vs. 21 ± 3 mg/day; *p* = 0.004).

### Response to medical therapy

**Figure 1:**
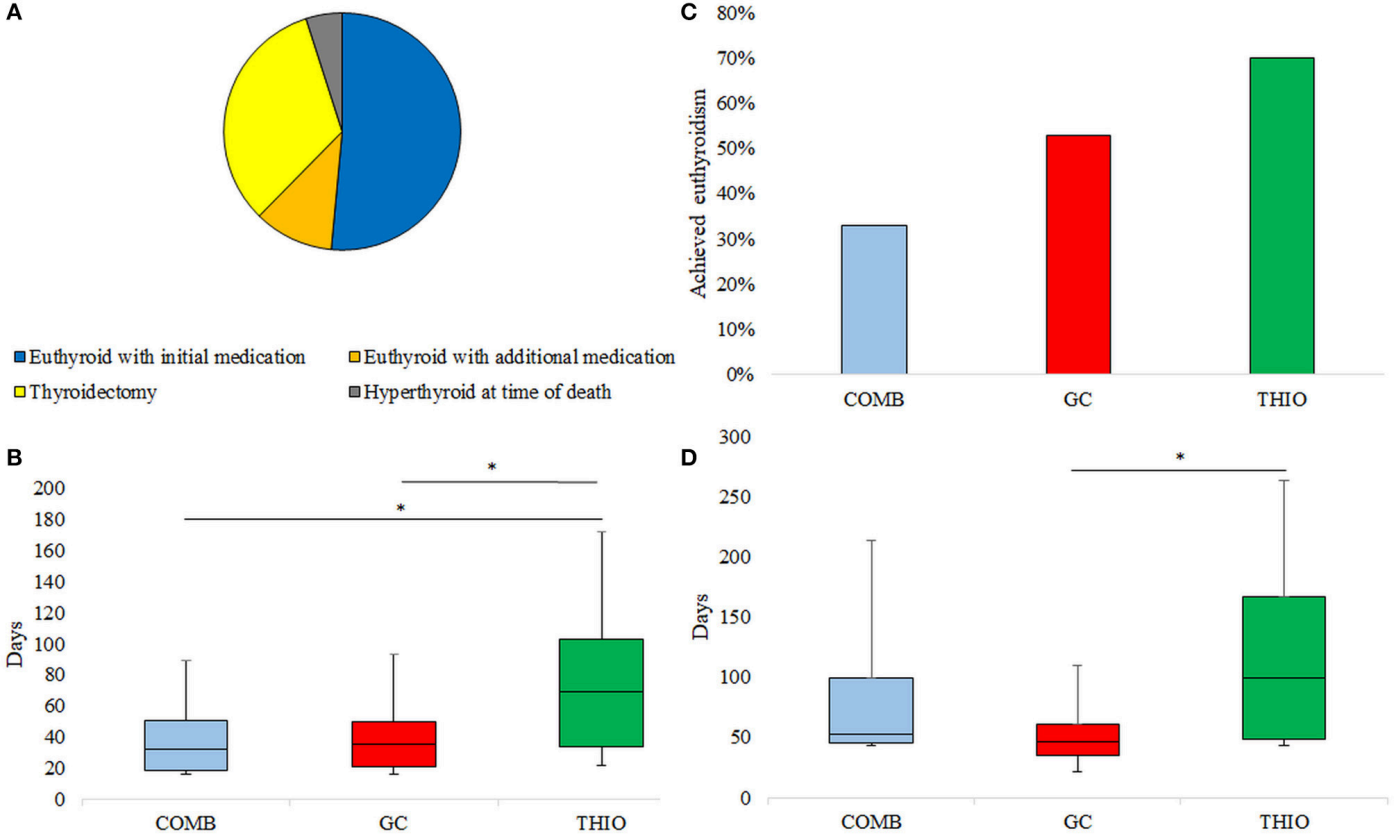
**(A)** Ultimate response to treatment of AIT, **(B)** Response rate to initial medication, **(C)** Time to normalisation of fT4 in those who responded to initial medication, **(D)** Time to euthyroidism in those who responded to initial medication. *p < 0.05.

Of the 64 patients treated for AIT, 33 (52%) became euthyroid with the initial treatment alone (Figure 1A). Rate of euthyroidism was highest in those initially treated with THIO (70%), followed by GC (53%) and COMB (33%); *p* = 0.045 (Figure 1B). Thirteen patients (7 GC and 6 THIO) subsequently transitioned to combination therapy at 35 (21–69) days; this achieved euthyroidism in 7 (11%), such that overall response rate to medical therapy was 63%. Two patients who became euthyroid with THIO were unable to cease medication due to recurrence of hyperthyroidism. They had successful radioiodine therapy for toxic nodular disease at 13 and 14 months post withdrawal of amiodarone.

Of those who became euthyroid with the initial medication choice, THIO patients took significantly longer to normalise fT4 than GC (69 [34–103] vs. 35 [21–50] days; *p* = 0.046) and COMB (32 [18–51] days; *p* = 0.04) (Figure 1C). Similarly, time to euthyroidism was 100 (49–167) days for THIO, 53 (45–99) days for COMB and 47 (35–61) days for GC (THIO vs. GC *p* = 0.01) (Figure 1D).

Response to medication was further assessed including only those who received the “recommended” medication at any point, either as mono- or combination therapy, that is, T1 treated with thionamides and T2 treated with glucocorticoids. Nine of 20 (45%) T1 patients treated with thionamides became euthyroid and 13 of 28 (46%) T2 patients treated with glucocorticoids achieved euthyroidism. Response rate to medication was similar when patients were classified according to CFDS alone: euthyroidism was achieved in 2/4 (50%) of patients with hypervascularity who received thionamides, and 15/33 (45%) with absent hypervascularity who received glucocorticoids. Compared with T2 patients who became euthyroid with glucocorticoids, T1 patients who became euthyroid with thionamides took longer to normalise fT4 (31 [29–85] days vs. 25 (19–51) days; *p* = 0.02) and TSH (102 [60–161] days vs. 47 (35–61) days; *p* = 0.049) after commencement of the relevant medication.

Twenty-one (33%) patients achieved euthyroidism only after thyroidectomy. Three patients (5%) had enduring AIT to the time of death from cardiac failure (*n* = 2) or sepsis (*n* = 1) despite combination medical therapy (Figure 1A).

### Thyroidectomy indications and complications

The characteristics of patients who underwent thyroidectomy and any perioperative complications are shown in Table 2. Twenty-one patients had thyroidectomy to treat AIT. The majority (71%) had initial treatment with COMB. Patients who underwent thyroidectomy demonstrated minimal response to medical therapy, with no significant difference between baseline and pre-operative fT4 (53.5 [31.9–>99.9] vs. 41.7 [30.1–80.3]; *p* = 0.65) or fT3 levels (7.1 [4.5–10.0] vs. 5.4 [4.0–13.5]; *p* = 0.58). Time to thyroidectomy following initial treatment was 49 (27–86) days in COMB, 62 (55–86) days in GC and 73 (47–192) days in THIO; *p* = 0.26). Time to thyroidectomy after initiation of recommended treatment (thionamides for T1 and glucocorticoids for T2) was 54 (37–88) and 66 (25–89) days, respectively (*p* = 0.64). Compared to those who responded to medical therapy, patients who underwent thyroidectomy were younger (54 ± 3 vs. 63 ± 2 years; *p* = 0.03) and tended to have higher initial fT4 (53.5 [31.9–>99.9] vs. 42.1 [31.5–58.1] pmol/L, though this was not statistically significant (*p* = 0.19) (Table 3). Nine of 15 (60%) of patients with initial fT4 ≥ 65 pmol/L ultimately required thyroidectomy to restore euthyroidism, compared to 12/36 (25%) of those with fT4 < 65 pmol/L (*p* = 0.01). Moreover, thyroidectomy patients tended to have higher prevalence of cardiac failure at baseline (81 vs. 58%; *p* = 0.09), including severely impaired left ventricular function (38 vs. 18%; *p* = 0.08) (Table 3). No patients had cardiorespiratory complications or death following thyroidectomy (Table 2), despite median ASA Physical Status classification 4.

**Table 2:** Characteristics of patients who underwent thyroidectomy and outcomes.

**Patient**	**Age range**	**Thyroid-ectomy indication**	**PreoperativefT4 (pmol/L), fT3 (pmol/L), TSH (mIU/L)^*†*^**	**Preoperative LVEF**	**Postoperative LVEF**	**ASA grade**	**Perioperative complications**
1	21–30	T	29.7, ND, <0.02	>60^*‡*^	>60	3	Nil
2	61–70	T, R	41.5, ND, <0.02	40	>60	3	Nil
3	71–80	T	36.0, ND, <0.02	60–65	>60	3	Nil
4	21–30	T	91.9, 10.2, <0.02	20^*‡*^	Tx	4	Nil
5	71–80	T, A, R	31.4, 4.2, <0.02	15–20	15–20	4	Neck haematoma (surgical evacuation); permanent hypoparathyroidism
6	31–40	T, H	68.7, 17.6, <0.02	70	70	4	Nil
7	61–70	T	>99.9, 21.2, <0.02	25	35–40	ND	ND
8	41–50	T, A	>99.9, 18.9, <0.02	40	40	3	Nil
9	71–80	T, A, R	25.3, 2.7, 0.09	25	25	4	Nil
10	31–40	T, A, R, H	50.5, ND, <0.02	15–20	Tx	4	Transient hypoparathyroidism
11	51–60	T, A	50.7, 16.7, <0.02	50–55	40–45	4	Nil
12	61–70	T, A	24.8, 3.2, 0.09	45–50	45–50	4	Nil
13	31–40	T, H	32.2, ND, <0.02	20–25	Tx	4	Nil
14	71–80	T	68.4, 8.7, <0.02	35	ND	4	Nil
15	61–70	T, R	41.7, 6.2, <0.02	60^*‡*^	50	3	Nil
16	41–50	T	>99.9, 41.3, <0.02	20	15–20	4	Nil
17	41–50	T	>99.9, ND, <0.02	60^*‡*^	65	ND	Nil
18	61–70	T, A	19.9, 3.7, <0.02	60	ND	4	Transient hypoparathyroidism
19	41–50	T	29.2, 5.4, <0.02	30	30	4	Nil
20	51–60	T, O	35.8, 5.3, 0.1	20–25	15–20	4	Nil
21	51–60	T, R	47.9, 4.3, <0.02	40–45	40	3	Nil
22	41–50	A	14.7, ND, 1.47	25^*‡*^	NA	4	Nil
23	61–70	A	22.6, ND, 1.50	20^*‡*^	NA	4	Nil
24	41–50	A	15.8, ND, 2.49	20^*‡*^	NA	4	Postoperative hypotension; acute kidney injury

*ASA, American Society of Anaesthesiologists; LVEF, left ventricular ejection fraction; ND, no data; NA, not applicable (euthyroid prior to thyroidectomy); Tx, cardiac transplant. Indications for thyroidectomy: T, thyrotoxicosis; A, future requirement for amiodarone; R, arrhythmia; H, worsening of heart failure; O, haemorrhage post fine needle biopsy of thyroid nodule resulting in airway compromise*.

† *Thyroid function tested at median 3.0 (0.5–10.5) days prior to thyroidectomy*.

‡ *These patients did not have transthoracic echocardiogram performed while thyrotoxic; LVEF was obtained from echocardiogram prior to the development of AIT*.

**Table 3:** Baseline characteristics of patients who responded to medical therapy vs. those who required thyroidectomy to restore euthyroidism.

	**AIT treated with thyroidectomy *n* = 21**	**AIT responded to medical therapy *n* = 40**	***p*-value**
Age (years)	53.6 ± 3.4	62.7 ± 2.4	0.03
Male sex	16 (76%)	33 (83%)	0.35
fT4 at diagnosis (pmol/L)	53.5 (31.9–>99.9)	42.1 (31.5–58.1)	0.19
fT3 at diagnosis (pmol/L)	7.0 (4.6–9.9)	7.5 (5.0–10.5)	0.68
Cardiac failure	17 (81%)	23 (58%)	0.09
LVEF <30%	8 (38%)	7 (18%)	0.08

An additional 3 patients had thyroidectomy after resolution of AIT, as amiodarone recommencement was considered essential for cardiac management and there was concern for AIT recurrence. One of these patients developed postoperative hypotension requiring treatment with dobutamine (Table 2). No patient received radioactive iodine ablation to facilitate amiodarone reintroduction.

### Thyroid histopathology and correlation with ultrasound

Of the 24 patients who underwent thyroidectomy, diagnosis was T1 AIT in 10, T2 in 13, and uncertain in 1 (due to lack of imaging) based on our classification.

However, histopathology showed evidence of T2 AIT (disrupted follicles, foamy histiocytes and fibrosis) in 23/24 thyroidectomy patients. Two patients with features of T2 also had significant nodular pathology, suggesting possible mixed disease. One patient had features of T1 AIT alone (multinodular goitre and Hürthle cell adenoma).

There was poor correlation between grey-scale sonography and histopathology: only 3/9 patients with sonographic goitre and/or nodules ≥10 mm had these findings confirmed histologically. In contrast, CFDS results were available for 19 thyroidectomy patients and had 100% correlation with histopathology: 18 patients with absent CFDS hypervascularity had histopathological evidence of T2, and 1 patient with hypervascular CFDS in a single nodule with absent parenchymal vascularity had mixed pathology confirmed on histopathology.

### Cardiac outcomes after treatment of AIT

Following restoration of euthyroidism, 60 patients had ongoing care at St Vincent's Hospital; duration of follow-up was 31 (15–60) months. During this time, 4/36 (11%) who did not have thyroidectomy recommenced (*n* = 3) or continued (*n* = 1) amiodarone; of these patients, 2 had T2, 1 had T1 and 1 had uncertain AIT type. Those who recommenced amiodarone did so for <1 week; no patient had recurrent AIT but follow-up after amiodarone reintroduction was of limited duration (range 2.5–7 months). The patient who continued amiodarone did not have recurrence of AIT during 12 months follow-up. Sixteen of the 24 (63%) thyroidectomy patients continued (*n* = 1) or recommenced (*n* = 15) amiodarone.

Thirteen patients had assessment of LVEF whilst thyrotoxic and within 1 year after thyroidectomy (Table 2). Two of these 13 had preserved LVEF even whilst thyrotoxic. Of the other 11, 2 (18%) had improvement in LVEF class post thyroidectomy (patients 2 [mild to normal] and 7 [severe to moderate]). Neither of these patients had reduced LVEF prior to development of AIT. Three patients underwent cardiac transplant within 1 year of thyroidectomy (Table 2); all had severely impaired left ventricular function (and one was already on the active cardiac transplant wait-list) prior to development of AIT.

## Discussion

This study evaluated the medical and surgical treatment of AIT in an Australian hospital with a cardiac transplantation service. The patients were at high risk of cardiac decompensation: 64% had a pre-existing diagnosis of cardiac failure and a further 11% had a history of cardiac transplantation. We found poor responses to medical treatment. In contrast, thyroidectomy under general anaesthesia executed by a team of experienced anaesthetists and with ICU monitoring was safe and effective despite presence of thyrotoxic state and underlying cardiac disease.

The recommendation that patients with T1 AIT be treated with thionamides and T2 with glucocorticoids was not followed for all patients in our series. This may be because treatment is frequently commenced before investigation results are available. However, it may also reflect the fact that clinicians are aware of the limitations of investigations used for classification of AIT and do not rely on them. For example, studies have shown very low or no 99Tm-sodium pertechnetate scintigraphic uptake in the majority of patients with both subtypes of AIT (8, 19), as was observed in our series. We found poor correlation between standard grey-scale sonography and histopathology, confirming that this investigation has low diagnostic value in AIT (8). Additionally, though CFDS is considered the investigation of choice in differentiating between T1 and T2 AIT (8), classifying AIT type by CFDS results alone did not improve medication response rates in our study. This is congruent with a previous Australian study, which found that CFDS was less predictive of treatment response compared with studies performed in iodine-deficient regions (19). These factors, along with the possibility of mixed/indeterminate forms of AIT, are likely to account for the high usage of initial combination thionamide and glucocorticoid therapy observed in our series. It is likely that patients treated with combination therapy from the outset were considered to be at higher risk of cardiac decompensation in the event that monotherapy was ineffective: they tended to have higher baseline fT4 levels and higher prevalence of heart failure.

We observed limited response to medical therapy, even when only those who received the recommended medication were considered. Furthermore, those who received initial combination therapy had the poorest response rate (33%), likely related to more severe thyrotoxicosis. Although highest response to initial therapy was observed in the thionamide group, these patients took the longest to achieve euthyroidism. In contrast, response to glucocorticoid was rapid: fT4 typically normalised by 1 month, as has been observed by other authors (19, 20).

Approximately one-third of patients in this series required thyroidectomy to restore euthyroidism. A high proportion (60%) of patients with severe thyrotoxicosis (fT4 ≥ 65 pmol/L) required thyroidectomy, consistent with reports that higher fT4 is associated with poorer response to medical therapy (20, 21). Traditionally there has been concern that thyroidectomy is associated with excess peri-operative risk in AIT due to the thyrotoxic state and underlying cardiac comorbidities (22). In a study of 33 patients undergoing thyroidectomy for AIT conducted at the Mayo Clinic (1985–2002), mortality was 9% (22). In contrast, we observed no surgical mortality despite a higher ASA score (4 vs. 3). The low rates of perioperative complications that we observed are in line with other recent series (13, 16, 21) and are particularly notable given 38% of patients undergoing thyroidectomy to treat AIT in our cohort had severely impaired left ventricular function even prior to development of thyrotoxicosis.

Unlike Tomisti et al. (16), we were unable to show improved cardiac function following thyroidectomy in those who had severe left ventricular systolic dysfunction while thyrotoxic. This is likely due to the fact that severe left ventricular dysfunction predated AIT in the majority of our cohort. Notably, the two patients who did show improvements in LVEF category post thyroidectomy had normal left ventricular function at baseline. Nevertheless, it is likely that the judicious use of thyroidectomy in our series contributed to the low mortality rate, which has previously been observed to be as high as 50% in patients with severely reduced LVEF (11). Moreover, thyroidectomy may permit cardiac transplant in those who would otherwise not be considered suitable due to presence of uncontrolled thyrotoxicosis.

Some patients in our series may have in time returned to euthyroidism without thyroidectomy; this is particularly true of those with T1 AIT. However, continued medical therapy carries the risk of medication-related adverse effects, and morbidity and mortality associated with protracted thyrotoxicosis (9–11). Additionally, thyroidectomy immediately obviates the risk of recurrent AIT after amiodarone reintroduction, which otherwise occurs in 73% initially diagnosed with T1 and 18% with T2 (23). Radioiodine ablation is an alternative option to prevent recurrent AIT (23, 24), but is unlikely to be successful for at least 6–12 months after amiodarone is ceased because the prolonged half-life of amiodarone results in persistent iodine load, and thus reduced radioiodine uptake (25). Furthermore, there is a risk of thyroiditis following radioiodine treatment, which may exacerbate the underlying cardiac condition.

This study is a retrospective audit and thus has several limitations. The frequency of thyroid function testing was variable and may have biased the recorded time to euthyroidism. Additionally, various assays were used for thyroid function testing, though we have corrected for this. Initial doses of medical therapy were not standardised and dose titration was at the discretion of the treating physician. Moreover, some clinicians may have had a lower threshold for adding a second medical therapy or proceeding to thyroidectomy. Though we observed no significant difference in thyroid hormone levels from baseline to time of surgery in those who underwent thyroidectomy, there was heterogeneity in this group. Nevertheless, a major strength of this study is that it provides a “real-world” report of the effectiveness of therapeutic strategies employed for AIT in a hospital specialising in cardiac transplantation, and is thus directly applicable to clinical practice.

In conclusion, we found that patients with AIT have a limited response to medical therapy despite specialist endocrine treatment in a tertiary hospital. This in part signifies the difficulties encountered when attempting to designate therapy by T1 or T2 status, but also reflects that prolonged treatment may be required to restore euthyroidism, with some cases of refractory AIT. In contrast, thyroidectomy is effective and safe when performed by an experienced surgeon in a centre with strong expertise in anaesthetising people with severe heart failure.

The ATA recommend that combined thionamide and glucocorticoid be used in patients who are deemed too unstable clinically to allow a trial of monotherapy (7). Whilst this approach is valid, our data shows that combination therapy has limited efficacy in the sickest patients. Hence, we agree with current recommendations that thyroidectomy be performed in patients with severe underlying cardiac disease, deteriorating cardiac function, or unresponsiveness to medication (6, 7). The majority of patients in our cohort who responded to glucocorticoids or thionamides had normal fT4 within 2 and 3 months, respectively. Therefore, it would be reasonable to consider a patient unresponsive to medication if fT4 remains substantially elevated beyond these timeframes. Based on our findings, we additionally suggest that clinicians have a low threshold for recommending thyroidectomy in patients who have cardiac failure, severe AIT (fT4 ≥ 65 pmol/L at diagnosis) and/or who require amiodarone on an ongoing basis, with the proviso that surgery only be undertaken in units with substantive anaesthetic cardiac failure expertise.

## Data availability statement

The raw data supporting the conclusions of this manuscript will be made available by the authors, without undue reservation, to any qualified researcher.

## Author contributions

JG and KS conceived the study. RB and DH were responsible for surgical and anaesthetic management, respectively. MI, MC, and HB reviewed the patient medical records. MI performed the data analysis. MI, KS, and JG contributed to the writing of the manuscript. All authors had the opportunity to review and edit the final manuscript.

### Conflict of interest statement

The authors declare that the research was conducted in the absence of any commercial or financial relationships that could be construed as a potential conflict of interest.
